# Proximity editing to identify RNAs in phase-separated RNA binding protein condensates

**DOI:** 10.1038/s41421-021-00288-9

**Published:** 2021-08-24

**Authors:** Guilong Zhou, Ruixia Niu, Yulu Zhou, Ming Luo, Yao Peng, Hui Wang, Zhao Wang, Guoyong Xu

**Affiliations:** State Key Laboratory of Hybrid Rice, Institute for Advanced Studies (IAS), Wuhan University, 430072 Wuhan, Hubei China

**Keywords:** Biological techniques, Cell death, Plant immunity

Dear Editor,

RNA binding proteins (RBPs) often phase separate RNAs into condensates to reprogram transcription and translation. Here, we find that catalytic domain of the *Drosophila* RNA-editing enzyme adenosine deaminase acting on RNA (ADAR) carrying E488Q mutation (HyperADARcd) outperforms other RNA editing enzymes and its RBP fusion allows proximity editing of dynamically associated RNAs in phase-separated condensates, e.g., during plant immune response in this study. This method will foster our understanding of the role of the interplay between RBPs and their RNA companions in cellular reprogramming through phase separation in any organism.

The interaction between RBPs and RNAs secures the correct flow of the genetic information and confers spatiotemporal reprogramming of cellular responses. This importance is highlighted by the presence of a large percentage of RBP candidate genes (> 6%) in the *Arabidopsis* genome^1^. However, many of them are annotated beyond nucleic acid-related functions (e.g., metabolism enzymes, transporters), leaving the immediate question: how to prove them as authentic RBPs? Certainly, the most straightforward way is to identify their RNA targets.

Although as the standard methods, ultraviolet crosslinking and immunoprecipitation-derived methods (CLIP; Supplementary Text S1) have not been widely used in plants and only two *Arabidopsis* RBPs have been reported, most likely due to their dependence on high-quality antibodies, low UV-cross linking efficiency, and particularly complex procedures^2–4^. This situation becomes even severer when RBPs are sensitive to the microenvironment and are prone to cluster or aggregate into condensates via phase separation either due to genetic mutation, developmental and environmental stimuli, or in vitro incompatible assay conditions^5,6^. Given the intrinsic multivalent interactions between RBP and RNA, RNA and RNA, and RBP and RBP, such a situation will be commonly encountered for most RBPs, for example, *Arabidopsis* AtUBP1c in this study.

AtUBP1c is a well-known RBP with three classical RNA recognition motifs (RRMs; Supplementary Text S2)^7^. In vitro purified yellow fluorescence protein-tagged AtUBP1c (YFP-AtUBP1c) underwent pronounced phase separation indicated by enhanced turbidity in the presence of the crowding agent that enhances intermolecular interaction, but failed to do so at a high salt concentration that disrupts such interaction (Fig. 1a–c). These typical phase separation behaviors granted the formation of dynamic condensates from the dilute phase that was promoted by transient overexpression of YFP-AtUBP1c to mimic the crowding condition in *N. benthamiana*. Interestingly, fluorescence recovery after photobleaching (FRAP) further demonstrated that these condensates became hardened and less dynamic with bare detection in the dilute phase upon activation of effector-triggered immunity (ETI; Supplementary Text S3) which was mediated by recognition between β-estradiol-controlled bacterial effector AvrPto and the corresponding constitutively expressed host target Pto (Fig. 1d, e; Supplementary Fig. S1a).

**Fig. 1:**
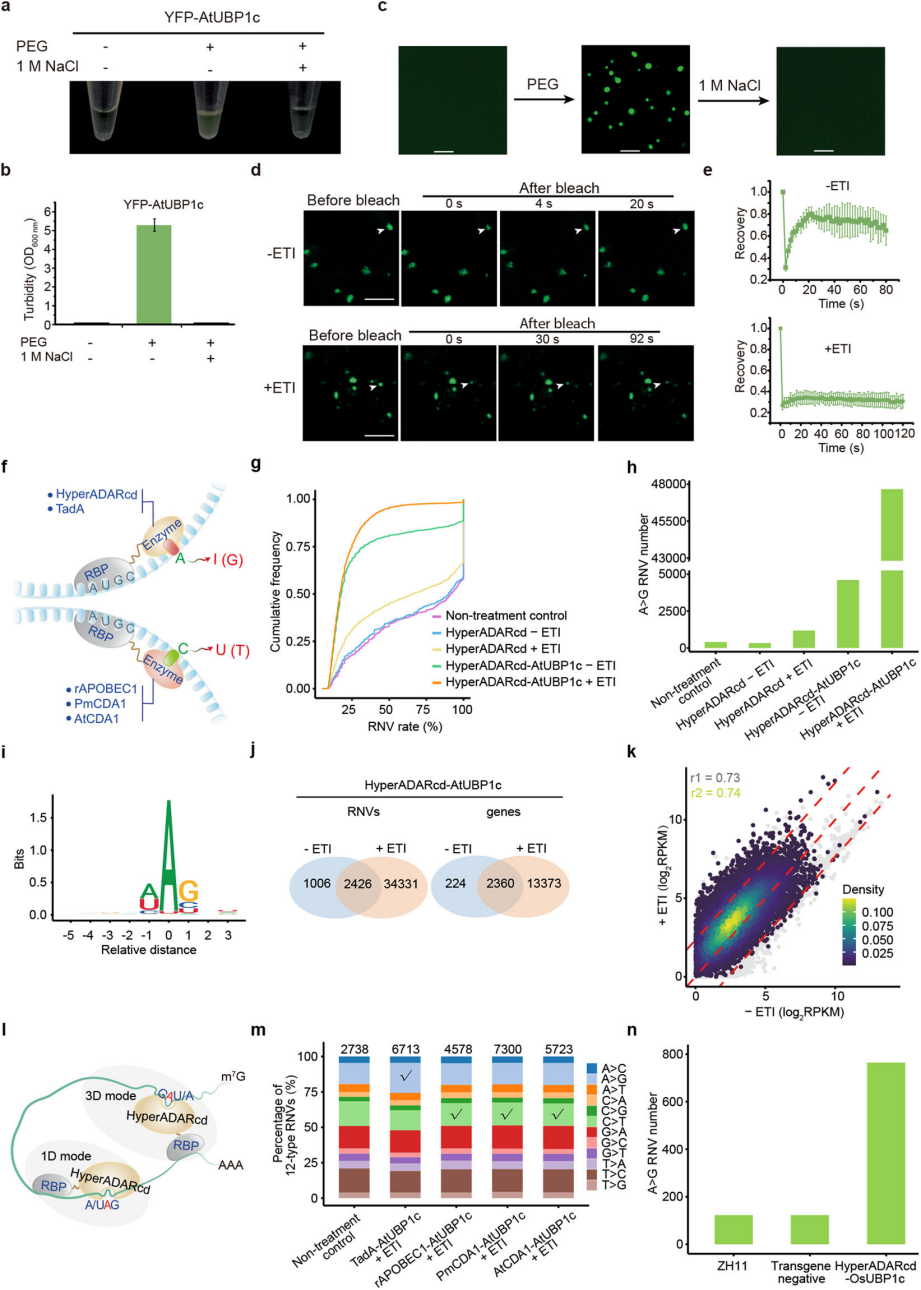
HyperADARcd outperforms other RNA editing enzymes in the identification of target RNAs in plants. **a–c** Examination of in vitro phase separation behaviors of purified YFP-AtUBP1c in the presence of crowding agent PEG with or without 1 M NaCl by white light photograph (**a**) and optical density measurement at 600 nm (OD_600 nm_; **b**) of turbidity changes and by confocal microscopy observation of droplet formation (**c**). Data in **b** are presented as means ± SD of OD_600 nm_ value in **a** (*n* = 8). **d**, **e** In vivo FRAP of YFP-AtUBP1c condensates without (−ETI; upper) or with (+ETI; lower) the induction of ETI through transient expression in *N. benthamiana*. Arrowhead indicates the region of interest for FRAP (**d**). Data in **e** are presented as means ± SD for the time course of the recovery after photobleaching in **d** (*n* = 6). Scale bar, 5 µm. **f** Schematic overview of RBP-directed adenosine deaminase (HyperADARcd and TadA) and cytidine deaminase (rAPOBEC1, PmCDA1, and AtCDA1) enzymes in conversion of nucleotide A to I (G) and C to U (T), respectively. **g–m** Identification of AtUBP1c target RNAs without (−ETI) or with (+ETI) ETI induction through transient expression of HyperADARcd or HyperADARcd-AtUBP1c in *N. benthamiana*. Non-treatment leaf serves as a control. **g** Cumulative frequency curve showing the RNV rate distribution patterns of non-filtered A>G RNVs. **h** The total number of filtered A>G RNVs (removing RNVs with rate > 90%). **i** Nucleotide context surrounding A>G RNVs in samples expressing HyperADARcd-AtUBP1c. *x*-axis, the relative distance from the editing sites (position 0). **j** Venn diagram showing constitutive and specific RNVs (left) and genes (right) of HyperADARcd-AtUBP1c without (−ETI) or with (+ETI) ETI induction. **k** 3D-density plot showing RNA levels of HyperADARcd-AtUBP1c+ETI target genes (13373 + 2360) in both conditions. Three red dashed lines from left to right indicate Z_log_2_RSfc (RNA-seq fold change) = 1.5, 0, and −1.5, respectively. Only genes with RPKM ≥ 1 in both conditions are shown as a background layer of gray dots. *r*1/*r*2, Pearson correlation coefficient for the total transcriptome and ETI target genes, respectively. **l** Schematic overview of 1D and 3D modes of RBP-directed HyperADARcd editing by linear and structurally spatial proximity, respectively. **m** AtUBP1c fusions with TadA, rAPOBEC1, PmCDA1, and AtCDA1 were used to detect target RNAs during ETI induction. Data are shown as percentages of the 12-type RNVs in each sample. Total RNV numbers are indicated above. The expected RNV type for each enzyme is checked. **n** Identification of OsUBP1c target RNAs in transgenic rice using HyperADARcd. Non-transgenic parent ZH11 and transgene-negative progeny are used as controls. Data are shown as filtered A>G RNV numbers. Each RNA-seq sample was combined from at least 9 individual plants. RNV numbers from different sample libraries were normalized using scale factors defined in Supplementary Fig. S2.

To capture the RNA companions in these condensates, we leveraged RNA editing enzymes whose RBP fusion allows proximity editing of RNA, leading to the detection of A>G and C>T variation in the target RNAs by adenosine deaminases and cytidine deaminases, respectively (Fig. 1f; Supplementary Table S1). Among these enzyme candidates, HyperADARcd has been successfully applied in the animal HyperTRIBE system^8,9^, was codon-optimized for *Arabidopsis* and fused with AtUBP1c. Prior to the occurrence of macroscopic cell death, we collected tissues of plants expressing HyperADARcd and HyperADARcd-AtUBP1c with (+ETI) and without (−ETI) ETI induction while non-treatment leaf served as a control (Supplementary Fig. S1b, c). Running our custom pipeline led to the finding that in comparison to non-treatment control, the distribution of RNA nucleotide variation (RNV) rate, namely editing efficiency that reflects the affinity of HyperADARcd to substrate nucleotides^9^, displayed different patterns only when HyperADARcd was fused to AtUBP1c (HyperADARcd-AtUBP1c vs HyperADARcd regardless of ETI induction) (Supplementary Figs. S2, S3a and Table S2). This characteristic of RBP-dependent RNA editing was also recapitulated by the huge increase of total RNV number, RNVs/RNA (RNV number per RNA molecule), and 3′ UTR enrichment (Supplementary Fig. S3b–d). Remarkably, the altered distribution patterns of RNV rate and increased RNV editing events were specific for A>G RNV, reminiscent of the editing specificity of HyperADARcd as an adenosine deaminase (Fig. 1g, h; Supplementary Fig. S3a, b). Analysis of the surrounding sequences of A>G RNVs showed a strong preference for nucleotide context (~99.55%; Fig. 1i). This preferential site can further serve as a more stringent filter to remove false-positive RNVs that might be due to endogenous nuclear A>I RNA editing if any, RNA-seq error, biallelic polymorphism, or unknown mechanisms underlying nucleotide differences between RNA and its encoding DNA.

The specificity of RBP-directed A>I RNA editing of this system allows further characterization of AtUBP1c’s behaviors on dynamic RNA binding in the condensates. In this scenario, we detected a dramatic increase of the total RNV numbers (36757 vs 3432) upon ETI induction (HyperADARcd-AtUBP1c/+ETI vs HyperADARcd-AtUBP1c/−ETI) and this increase was due to more bound mRNA targets (15,733 vs 2584) plus higher RNVs/RNA (median, 2 vs 1) (Fig. 1j; Supplementary Fig. S4a). Most of the 15,733 HyperADARcd-AtUBP1c/+ETI targets (84.21%) were well expressed (RPKM ≥ 1) and maintained at a steady RNA level (|Z_log2RSfc | < 1.5; RSfc, RNA-seq fold change) in both conditions. These target genes were enriched for GO terms related to normal metabolism, catabolism, and transport (Fig. 1k; Supplementary Fig. S4b, c and Table S3f). This increased editing was not chaotic but in an ordered manner since ETI induction only caused minor changes of editing on different RNA features but not the alternation of overall RNV rate (Supplementary Figs. S3c, S4d, e). Otherwise, messy RBP binding would have led to random editing on nucleotide A, and thus RNV numbers should have been proportional to the ratios of nucleotide A content or HyperADARcd preferential editing sites for each feature, which was not observed in our results (Supplementary Fig. S4e).

Interestingly, RNVs were more frequently found in 3′ UTRs in plants expressing HyperADARcd with or without ETI induction when compared to non-treatment plant control (Supplementary Figs. S3d, S4e), which is consistent with the binding preference of AtUBP1c in *Arabidopsis*^7^ and its *Nicotiana plumbaginifolia* homolog. RBPs usually selectively bind to RNA targets through recognition of a continuous binding sequence. To illustrate the spatial relationship between RNVs and RBP binding sites, we proposed a one-dimensional (1D) editing mode to describe RNVs by linear proximity editing and a three-dimensional (3D) editing mode that shows structurally spatial proximity editing (Fig. 1l). Because of the failure of detection of a consensus motif from a range of RNV surrounding sequences, we believed that most RNVs were derived from 3D editing, which is consistent with the observation in other systems^9^. Indeed, upon ETI activation, RNVs/RNA increased and was better correlated with target RNA length (Supplementary Fig. S4f). This was further implied by another finding that the constitutive RNA targets during ETI had the highest RNVs/RNA and RNV rate (Supplementary Fig. S4g, h). We designed three control experiments and further demonstrated the reproducibility of HyperADARcd system in plants (Supplementary Figs. S5–S7, Text S4 and Tables S4, S5). We also explained the observation of some occasional T-C RNVs (Supplementary Fig. S6b and Text S5).

In an attempt to find alternates, we in parallel compared other four editing enzymes with HyperADARcd upon ETI induction, including adenosine deaminase TadA and cytidine deaminases rAPOBEC1, PmCDA1, and AtCDA1, but found that none of their AtUBP1c fusions could effectively edit the target genes (Fig. 1m; Supplementary Fig. S8 and Table S2). Therefore, HyperADARcd outperformed other RNA-editing enzymes in transient expression systems and transgenic *Arabidopsis*. We then proceeded to examine the function of *Oryza sativa* homolog of AtUBP1c, OsUBP1c (HyperADARcd-OsUBP1c), in crops. Compared to the non-transgenic parent and transgene-negative progeny, the transgene-positive plants showed obviously increased A>G RNV number (Fig. 1n; Supplementary Fig. S9). It is worth noting that our pipeline recommends filtering RNV with a rate above 90% due to inherent sequence discrepancy between lab stocks and the reference genome, which allows more sensitive detection of editing events for species with higher genomic diversity, such as rice in this study (Supplementary Fig. S9c–e).

Global profiling of the RBP binding spectrum is an essential part of the human Encyclopedia of DNA Elements project, and great progress has been made in studies of both methodology and mechanism. Our whole toolkit and case studies here will assist to fill these gaps and pave the way for understanding the interplay between RBPs and RNA targets involved in cellular reprogramming of plant development and stress responses^10^. HyperADARcd is still under development and requires extensive improvements, such as using controllable systems (e.g., β-estradiol; Supplementary Fig. S10), lowering the editing preference, and increasing editing efficiency (Supplementary Text S6). Since the phase separation behavior is rooted in the intrinsic multivalent interaction among RBPs and RNAs, our method could be faithfully harnessed in other organisms to resolve the shared issues.

## Supplementary information

Supplementary figures and texts

Supplementary Table S1

Supplementary Table S2

Supplementary Table S3

Supplementary Table S4

Supplementary Table S5

Supplementary Table S6

Supplementary Table S7
