# Supplementary material for: Proximity editing to identify RNAs in phase-separated RNA binding protein condensates
Source: Cell Discov. 2021 Aug 24;7:72. doi: 10.1038/s41421-021-00288-9 (PMC8382829; doi:10.1038/s41421-021-00288-9)
Supplement: Supplementary file 1 — Supplementary figures and texts [file 41421_2021_288_MOESM1_ESM.pdf]

Supplementary Information

Materials and methods 1

Supplementary Text S1: Identification of RBP-associated target RNAs *in vivo* 5

Supplementary Text S2: Background of AtUBP1c 7

Supplementary Text S3: Background of effector-triggered immunity 7

Supplementary Text S4: Reproducibility of HyperADARcd system 8

Supplementary Text S5: Explanation of T>C variation detected in HyperADARcd results 9

Supplementary Text S6: Perspective on HyperADARcd system 10

Supplementary Text S7: Zero-background ligation-independent cloning of RBP fusion with HyperADARcd 12

Supplementary Text S8: References 14

Supplementary Figures 18

Materials and methods

Plasmid construction

HyperADARcd, TadA, rAPOBEC1, PmCDA1 and AtCDA1 were either synthesized or amplified from other plasmids or cDNA (Supplementary Table S1). Arabidopsis AtUBP1c (AT3G14100.1), AtMPK3 (AT3G45640.1) and AtGRP7 (AT2G21660.1), and rice OsUBP1c (Os11g40510.1) were amplified from *Arabidopsis thaliana* Col-0 and Nipponbare cDNA, respectively. Zero-background ligation-independent cloning procedure for constitutive and inducible expression of RBP-HyperADARcd are detailed in Supplementary Text S7. Briefly, forward and reverse primers for RBPs were flanked with different adaptor sequences according to the desirable conformations. Regular PCR was done with any commercial high-fidelity DNA polymerase. PCR products were purified using any commercial gel-purification kit. Purified PCR products were submitted for LIC treatment in a 10 μl reaction using T4 DNA polymerase (NEB, M0203) in the presence of dATP. In parallel, the chosen vector was linearized using ApaI followed with LIC treatment in a 100 μl reaction using T4 DNA polymerase (NEB, M0203), instead, in the presence of dTTP. LIC reaction was performed at 12 ℃ for 30 min and inactivated at 75 ℃ for 20 min. 2 μl each of LIC>Treated PCR fragment and vector were co-incubated at 75 ℃ for 5 min followed with an extra incubation at 22 ℃ for 10

min. The remaining vector was saved at -20 °C. The resulting ligation products were used to transfer any ccdB sensitive *E.coli* strain, such as TOP10 and DH5α. Regular molecular cloning is followed for the rest of the steps.

HyperADARcd-related plasmid information has been deposited in NCBI (Supplementary Fig. S10 and Table S6). All constructs used in this study are listed in Supplementary Table S6 and will be available upon request.

Protein expression and purification in *E.coli*

MBP-YFP-AtUBP1c fusion protein was expressed in *E. coli Rossetta* (DE3) cells (TransGen, CD801-02). Cells were cultured to $OD_{600\ nm}$ = 1.0 for 6 h at 25 °C in terrific broth supplied with carbencillin (50 mg l^{-1}) and further grown for another 18 h at 16 °C in the presence of 0.3 mM isopropyl β-D-1-thiogalactopyranoside (IPTG). Cells were then spun down, resuspended in lysis buffer (20 mM Tris-HCl pH 7.4, 1 mM EDTA, 500 mM NaCl, 10 mM 2-Mercaptoethanol, 1 mM PMSF), and lysed with a high-pressure homogenizer (ATS Engineering, FB-110X). The cell lysate was centrifuged at 15,000 g for 1 h at 4 °C. The fusion protein in the soluble supernatant was purified with amylose resin (NEB, E8021S) in an ultrafiltration centrifugal column (Millipore, UFC801008) according to the manufacturer's instructions. MBP tag was removed by incubation with 10 μg/ml factor Xa protease (NEB, P8010S) overnight at 23°C. Protein concentration was measured using Bradford-based Easy Protein Quantitative Kit (TransGen, DQ101-01) and adjusted to 0.5 μM. Proteins in each step were examined by SDS-PAGE through coomassie brilliant blue staining. Purified protein was flash-frozen in liquid nitrogen and stored in the storage buffer (20 mM Tris-HCl pH 7.4, 1 mM EDTA, 50 mM NaCl) at -80 °C until use.

***In vitro* phase separation assay**

In vitro phase separation assay was performed with 0.5 μM purified protein in the storage buffer. Phase separation was induced by adding PEG8000 (Sigma, BCCC7539) at a final concentration of 12% (w/v) or reversed by adding NaCl at a final concentration of 1 M. Samples were dropped to a confocal dish and observed with Leica TCS SP8 upright microscopy equipped with 63× oil immersion objective using a 514 nm laser for excitation and 517–557 nm filter for emission. Turbidity was measured as optical density at 600 nm with 1 μl sample using NanoDrop One Microvolume UV-Vis Spectrophotometer.

***N. benthamiana* growth and transient expression**

N. benthamiana plants were grown on soil (Pindstrup, Denmark) at 22 °C under 12/12-h light/dark cycles with 55% relative humidity. Binary T-DNA vectors were transformed into the *Agrobacterium* strain GV3101 (pMP90). Colony PCR positive transformant was cultured overnight at 28 °C in Luria-Bertani broth supplied with kanamycin (50 mg l^{-1}), gentamycin (50 mg l^{-1}), and rifampicin (25 mg l^{-1}). Agrobacterial cells were

spun down at 2,600 g for 5 min and resuspended in infiltration buffer (10 mM 2-(N-morpholino) ethanesulfonic acid (MES), 10 mM $MgCl_2$, 200 μM acetosyringone). Cell density was adjusted to $OD_{600\ nm}$ = 0.1 and incubated at room temperature for 4 h before infiltration. Different agrobacteria were equally mixed and infiltrated on 3-week-old plants with 1 ml needleless syringes.

Agrobacterium 35S::Pto (pWZ37) expresses the plant target of pathogen effector AvrPto whose expression is induced by β-estradiol from *Agrobacterium* estradiol::AvrPto (pWZ46). These two *Agrobacteria* were mixed with *Agrobacteria* expressing HyperADARcd and HyperADARcd-AtUBP1c, respectively, and plants were infiltrated with different mixtures. 40 hours post infiltration (hpi), sterile water (-ETI) or 50 μmol β-estradiol (+ETI; Sigma, #E8875) were sprayed on HyperADARcd- and HyperADARcd-AtUBP1c-contained leaves. In addition, *Agrobacteria* 35S::Pto and estradiol::AvrPto combination was mixed with *Agrobacteria* containing TadA-AtUBP1c, rAPOBEC1-AtUBP1c, PmCDA1-AtUBP1c and AtCDA1-AtUBP1c, respectively and leaves were sprayed with 50 μmol β-estradiol. All samples were collected 10 hours after treatment. In the meantime, equal amount of non-infiltrated leaves were collected as the Non-treatment control. *Agrobacterium* containing YFP-AtUBP1c was co-infiltrated with *Agrobacteria* 35S::Pto and estradiol::AvrPto combination and the leaves with/without ETI induction were instead subjected to FRAP assay.

Arabidopsis transformation and quantitative PCR

Arabidopsis transformation and quantitative PCR were performed exactly as described previously[1]. 50 μmol β-estradiol was infiltrated into Arabidopsis leaves. Quantitative PCR primers for HyperADARcd and hygromycin resistance gene are included in Supplementary Table S6.

FRAP

FRAP was performed on a two-photon laser scanning fluorescence confocal microscopy (Leica, TCS SP8) equipped with a 40× objective lens. The region of interest was bleached using a 514 nm laser pulse (two iterations, 60% intensity). Recovery was recorded every two seconds for a total of 80 s (YFP-AtUBP1c without ETI) or 120 s (YFP-AtUBP1c with ETI) after bleaching. The recovery curves were analyzed using Origin 9.

Rice growth and transformation

Binary T-DNA HyperADARcd-OsUBP1c vector was transformed into the *Agrobacterium* strain EHA105 (pEHA105) and introduced into *O. sativa* cultivar ZH11. For progenies that were grown on the hygromycin (25 mg l^{-1}) selection medium, PCR confirmation of the transgene ADARcd-OsUBP1c was performed. PCR negative progenies and non-transgenic ZH11 parent were used as controls.

RNA-seq library preparation and analysis

About 200 mg leaf tissue was ground with liquid nitrogen and further homogenized in TRIzol Reagent (Invitrogen, #15596018). A total amount of 1.5 μg RNA per sample was used for oligo(dT)-magnetic bead-based purification of polyA-contained RNA (Invitrogen, #61002). RNA-seq libraries were made with NEBNext® Ultra™ RNA Library Prep Kit for Illumina® (NEB, #E7770). All procedures followed each manufactural instruction. Illumina PE150 sequencing was run by Novogene company on the NovaSeq 6000 platform. RNA-seq analysis and detection of differential expression genes using Z_score method was performed as described previously[1].

Bioinformatics

Nb-1 reference genome v1.0.1 and gene annotation file Niben101_annotation.gene_models.gff were used for *N. benthamiana* analysis. Nipponbare MSU V7 and gene annotation file version_7.0 all.gff were used for rice analysis. *A. thaliana* Col-0 TAIR10 reference genome and gene annotation file version Ensemble V39 were used for Arabidopsis analysis. The workflow of RNV detection pipeline is depicted in Supplementary Fig. S2. Generally, users unzip the downloaded pipeline from our website (http://uorflight.whu.edu.cn/public/) to their working directory and follow the user guide in Supplementary Fig. S2 to automatically obtain the result tables for self-tailored figure preparation. The genomic sequence of 5 bp flanking the A>G RNV sites were used for consensus sequence enrichment analysis in the MEME suite. The matrix returned by MEME was input to FIMO to identify HyperADARcd preferential site distribution on mRNA features of the whole representative transcripts. GO term information of all *N.benthamiana* genes was extracted from Niben101_annotation.gene_models.gff using key word "Ontology_term" and was further grouped into three namespaces of "biological_process" (P), "molecular_function" (F) and "cellular_component" (C) using go-basic-obo file downloaded from Gene Ontology website. GO term information of all Arabidopsis genes was directly subset from ATH_GO_GOSLIM.txt downloaded from TAIR

(https://www.arabidopsis.org/download_files/GO_and_PO_Annotations/Gene_Ontology_Annotations/ATH_GO_GOSLIM.txt). GO information was tidied into .txt file with four columns of gene_ID, GO identity, GO namespaces and the source of GO term. The information used in our study is included in the Supplementary Table S7. GO enrichment analysis was performed with the GO information file using clusterProfiler package in R.

The RNA-seq data in this study have been deposited in Gene Expression Omnibus under accession number PRJNA664696. All figures presented in this manuscript except Supplementary Figs. S2 and S10 are associated with these data. Custom code in this study is integrated into RNV detection pipeline that can be downloaded from our database website (http://uorflight.whu.edu.cn/public/).

Statistical methods

For non-parametric statistics, Wilcoxon rank-sum test and Kruskal-Wallis test were used for two and multiple comparisons followed by post-hoc analysis with Dunn's test, respectively. Pearson's chi-squared test was used for comparisons of the frequency distribution. Statistical tests were performed in RStudio with $P > 0.05$ not significant, $^{*}P < 0.05$, $^{**}P < 0.01$, $^{***}P < 0.001$ and $^{****}P < 0.0001$. Other information about statistical parameters can be found in figure legends.

Supplementary Text S1: Identification of RBP-associated target RNAs *in vivo*

Identification of associated RNAs for a given RBP is central and fundamental to investigate the physiological roles of RBP-RNA interaction. Most if not all available methods contain four main steps, sequentially as marking, purifying, constructing and reading (MPCR) their interaction. The step of marking involves covalent crosslinking, such as using reversible formaldehyde and non-reversible ultraviolet (UV). After marking, the RBP-RNA adduct of interest is purified through immunoprecipitation with antibodies against either endogenous RBPs or epitopes that are tagged with RBPs. Immediately, the bound RNA are constructed into RNA-seq libraries through variable protocols. The resultant libraries are sequenced on NGS platforms and target RNAs are detected using different bioinformatic pipelines. RNA>Immunoprecipitation (RIP) features formaldehyde crosslinking without RNase digestion, and UV crosslinking and immunoprecipitation (CLIP) features UV crosslinking with RNase digestion. When the CLIP protocol involves feeding cells with photoreactive 4-thiouridine (4SU) into nascent RNAs to improve UV-crosslinking, this derivate is called PAR-CLIP[2]. Other CLIP-based derivative methods are variables of strategies in the purifying and constructing steps, including HITS-CLIP[3], iCLIP[4], BrdU-CLIP[5], Fast-iCLIP[6], irCLIP[7], eCLIP[8], sCLIP[9], GoldCLIP[10]. Readers are highly encouraged to read recent excellent reviews on advantages and disadvantages of each method[11-13].

Here we briefly mention general considerations of those methods particularly on their implementation in plants from aspects of false-positive and false-negative rates. It was reported that RIP-seq usually yielded high percentage of target RNAs out of the whole transcriptome and suffered poor reproducibility in animal systems, implying high rates of false-positive and false-negative rates[14]. This is likely because formaldehyde crosslinks not only RBP-RNA but also proximal interacting RBPs in the RNP complex. Therefore, the sequencing results only compromises a subset of RNAs belonging to the RBP of interest. Meanwhile, non-specific RNAs in the cell lysate likely reassociate with IP complex and constitute another source of false positive[15,16]. To remedy this non-specific issue, CLIP-based methods use UV crosslinking which has high preference for protein-nucleic acids. The non-specific bound RNA fragments on antibodies are largely separated from crosslinked RBP-RNA adduct by running SDS-PAGE gel (except for Fast-iCLIP, sCLIP and GoldCLIP) though they could not be completely omitted[17]. Target RNA fragments in RBP-RNA adduct are

specifically retrieved by cutting western blotting regions which immigrate slower than RBP alone because of RNA association. However, CLIPs capture a snapshot of RBP-RNA interaction, many of which are weak and transient interactions. It remains unknown whether these interactions are biological meaningful or that they are actually false positive[14,18].

Nevertheless, CLIPs are superior to resolving the false positive issue when further coupled with enrichment computation against input controls of the total transcriptome or unwashed IP RNAs[8,19]. Along with another evident advantage of identifying RBP binding regions, CLIPs have become the current gold standard for detection of target RNAs *in vivo* for a given RBP. But these methods still have the false-negative issues that usually arise from low crosslinking efficiency, low antibody quality and a big RNA loss during complex processing procedures. It is estimated that UV-crosslinking generally has 1%-5% efficiency[20], has a bias crosslinking toward pyrimidines, especially uridines, and could not efficiently crosslink double stranded RNAs either[11,21,22]. These issues become more serious in plants because they have rich secondary metabolites that prevent the UV penetration and absorption. In addition, the quality of antibodies is a prerequisite for a successful CLIP. Even working with qualified antibodies causes 18% failure in a standard large-scale CLIP assay[19]. Lastly, the complex procedures cause degradation or loss of target RNA fragments during multiple rounds of purification. All these issues necessitate the requirement of large amount of starting materials. In many cases, this requirement cannot be easily met, e.g., root stem cells in plants.

Recently, eCLIP has been benchmarked and successfully used to profile 150 RBPs in animal cells[19]. This large-scale CLIP mapping of RBPs started with the identification of a RBP-specific and IP-grade antibody. Screening over 700 antibodies identified 438 such qualified antibodies, of which only 82% worked successfully to the middle point of the whole procedure, IP-western blot. Ultimately, after a complex procedure 223 high quality data sets were obtained from 488 initial eCLIP experiments (45.7% success rate). Average percentage of uniquely genomic mapped reads lies within 0.01%-0.8% (mean ± s.d., 0.33% ± 0.16%), which means most of the sequencing reads are useless. Most likely, these shortcomings, particularly low crosslinking efficiency, high quality antibody-dependence and complex procedures, become the most compelling reasons for the fact that so far only two plant RBPs have been reported using this method[11].

As a parallel strategy, marking RBP-RNA interaction *in vivo* by RNA enzymes has been reported. It labels target RNAs without crosslinking and avoids the IP purification of the RBP-RNA complex. The RNA enzyme used in RNA-tagging technology is the *Caenorhabditis elegans* poly(U) polymerase PUP-2 which catalyzes the addition of a string of uridines (the U-tag) to target RNAs when expressed as a fusion protein with the RBP of interest[23]. Reading RBP-RNA interaction through NGS could be achieved through selective sequencing of the poly(U)-tagged RNA species or through whole transcriptome sequencing. It is noted that the former method requires a special library construction protocol. Using this tagging system, subcellular recording mRNA localization has been reported by fusion of PUP-2 with organelle marker proteins, similar

to the application of APEX-seq in the investigation of the spatial transcriptome[24,25]. TRIBE (targets of RNA-binding proteins identified by editing) used ADARcd (catalytic domain of the *Drosophila* RNA-editing enzyme ADAR) to mark target RNAs by catalyzing adenine to inosine editing when directed to RNAs by the fused RBP of interest[14]. A new version of TRIBE, namely HyperTRIBE which harnesses a hyperactive mutation E488Q of the ADARcd (HyperADARcd), largely increases the editing efficiency of this system[26]. Albeit RNA-enzyme-based marking strategy could not detect the binding motif in comparison to CLIPs, it is evident that this strategy is procedure-simple, cost-effective and likely of lower false-positive rate[17]. With continuing improvement, it will become prevailing to provide a complement strategy with CLIPs for understanding RBP-RNA interaction.

Supplementary Text S2: Background of AtUBP1c

AtUBP1c stands for oligouridylate-binding protein 1c, and has another two family members, UBP1a and UBP1b in Arabidopsis. They share high protein sequence identity with human T cell intracellular antigen-1 (TIA-1) and its family member TIA-like 1 (TIAL1), orthologues of which can be found from yeasts to metazoans[27]. They contain three canonical RNA binding domains, RNA recognition motif (RRM), and individual RRM of human TIA1 and TIAL1 has different RNA binding specificities[28]. Subcellular localization reveals that both human and Arabidopsis orthologues are found in the nucleus and cytosol. In the nucleus, human TIA1 and *Nicotiana plumbaginifolia* orthologue UBP1 participate in mRNA splicing by binding to U-rich intron sequences[29-31]. With the similar binding preference, in the cytosol, human TIA1 and TIAL1 were also found to bind U-rich sequences within 3' UTR to sequester mRNAs into stress granules[32-34].

Human TIA1 and TIAL1 have been reported to regulate many physiological processes, including cell cycle progression, inflammation and apoptosis[33]. In plants, AtUBP1b was used as a marker of the stress granule and its formation is responsive to heat stress[35]. Overexpression of AtUBP1b led to enhanced heat tolerance as well as ABA hypersensitivity[36,37]. AtUBP1c was also localized to stress granules upon hypoxia stress and a loss of function mutant showed decreased survival rate when treated by hypoxia[32]. In this study, we used our lab stock AtUBP1c which was originally used as a stress granule marker, to demonstrate its capacity of phase separation *in vivo* and *in vitro*, and further illustrated the efficiency of HyperADARcd in the identification of mRNA components in its phase separation condensates.

Supplementary Text S3: Background of effector-triggered immunity

Plants share similar mechanisms with animals to detect non-self pathogenic signals for immunity establishment. They use cell surface-localized transmembrane receptors, pattern recognition receptors (PRRs) to detect molecular patterns from both pathogens and damaged hosts, consequences of which activate robust pattern-triggered immunity (PTI)[38-41]. Plants also have intracellular receptors, nucleotide-binding leucine-rich

repeat proteins (NLRs), to directly or indirectly detect pathogen effectors, leading to the activation of effector-triggered immunity (ETI)[42,43]. These NLRs usually contain N terminals of either coiled-coil (CC) domain in CC type NLRs (CC-NLRs), or Toll/interleukin-1 receptor (TIR) domain in TIR type NLRs (TIR-NLRs). A number of sensor NLRs from both types require helper NLRs (i.e., ADR1, NRG1 and NRC1) to transduce recognition signals through EDS1 modules, downstream of which includes boosting resistance activity and often causing hypersensitive cell death[43]. Recent studies show that these two layers of immunity mutually potentiate each other to achieve full and effective resistance against bacteria, mechanistically answering the long observation of the blurred PTI-ETI dichotomy[44-46].

In the indirect recognition manner, the sensor NLRs can detect the effector-mediated modification of the guardee or decoy[42]. According to this mode, Pto used in this study is a decoy from tomato whose kinase activity is monitored by NLR, Prf protein. Prf senses changes upon the interaction between Pto and *Pseudomonas syringae* pv. *tomato*-derived effector, AvrPto[47-49]. Transient expression of Pto-AvrPto pair in *N. benthamiana* could faithfully elicit a rapid hypersensitive cell death and has been widely used to study ETI-triggered cell death[50]. In our study, we used CaMV 35S promoter to constitutively express Pto but used β-estradiol to induce the expression of AvrPto, through which we can control the timing of ETI activation and collect tissue before the occurrence of macroscopic cell death.

Supplementary Text S4: Reproducibility of HyperADARcd system

Reproducibility of HyperADARcd system has been extensively validated in the original development in animals[14,26]. To further prove it in plants, we designed three control experiments (Supplementary Figs. S5-7, Tables S4, S5). First, we found that three independent experimental replicates show 60-80% overlapping of target RNAs. Second, HyperADARcd-AtUBP1c in transgenic Arabidopsis shares 76.51% identity with AtUBP1c target RNAs generated by RNA>Immunoprecipitation method. Third, RNV detection with another RBP protein, AtGRP7 also specifically detected its target RNAs while a non-RBP protein, AtMPK3 can not. Detailed information is as following:

First, we repeated the RNV detection of HyperADARcd-AtUBP1c upon ETI activation sample in *N. benthamiana* in two independent experiments (Rep2/3) and compared them with the first experiment result (Rep1). We found that all three replicates displayed almost the same distribution of RNV rate (Supplementary Fig. S5a, Table S4). Even though Rep2/3 detected fewer A>G RNVs and genes, approx. 60% target RNAs occurred in all three replicates and this number climbed approx. 80% in any two replicates (Supplementary Fig. S5b-e). The reproducibility of HyperADARcd system is further demonstrated by the observation that the overlapped A>G RNVs and genes displayed mild changes with different RNV rate thresholds (Supplementary Fig. S5f). In sum, the three independent experiments demonstrate the reproducibility of this system.

Second, we generated transgenic Arabidopsis expressing HyperADARcd-AtUBP1c fusion protein under the control of β-estradiol and intersected their target RNAs with a previous RIP result of AtUBP1c. Similar to the observation of HyperADARcd-AtUBP1c without ETI sample in *N. benthamiana*, we found that RNVs in Arabidopsis HyperADARcd-AtUBP1c also showed A>G specific detection (Supplementary Fig. S6a, Table S5). However, we observed fewer numbers of A>G RNVs and target genes in transgenic Arabidopsis (Supplementary Fig. S6c). GO analysis found that these target genes participated in different catabolic processes, senescence and aging (Supplementary Fig. S6d and Table S5). We further compared our target RNAs with the whole list of AtUBP1c targets which were detected using RIP method following with microarray detection (RIP-chip). The publicly available RIP-chip list contains all potential AtUBP1c RNA targets identified under the conditions of light, dark, hypoxia and reoxygenation treatments[32]. Our target RNAs only take a small part of it (7.27%, 1042/14325), suggesting that AtUBP1c binds different RNA pools to be adaptive to changed cellular status. Most importantly, we found that 76.51% of target genes (1042/1362) detected by HyperADARcd system occurred in the RIP-chip result, demonstrating a high reproducibility of this system (Supplementary Fig. S6e).

Third, we verified the reproducibility by examination of another RBP, AtGRP7 through comparing it with a non-RBP, AtMPK3 in transgenic Arabidopsis. We used β-estradiol to induce the expression of AtGRP7-HyperADARcd and AtMPK3-HyperADARcd. As expected, RNV detection found tens of fold changes of both A>G RNV and target gene numbers for AtGRP7 (Supplementary Fig. S7a, b). Interesting, these genes were involved in the biological functions of protein folding and refolding as indicated by GO analysis (Supplementary Fig. S7c). Collectively, the above three control experiments clearly validate the reproductivity of the HyperADARcd system in plants.

Supplementary Text S5: Explanation of T>C variation detected in HyperADARcd results

HyperADARcd functions as an adenosine deaminase which edits adenosine (A) to inosine (I) on mRNAs, leading to A>G variation in the final sequencing results. However, our RNV detection also found many T>C variation (Supplementary Fig. S3b, 6a). This is due to the non-stranded RNA-sequencing used in our library construction that cannot tell the directions of the sequencing read relative to the annotated mRNA. Since antisense transcripts exist in the transcriptome, their editing by HyperADARcd leads to A>G RNV in the antisense transcripts. However, antisense transcripts are not well annotated in the current annotation files. Thus, A>G edited antisense reads will give rise to T>C in the annotated sense transcript that will be recorded in the RNV detection pipeline as depicted (Supplementary Fig. S6b). This could be improved by construction of a stranded RNA-seq library which costs more than a regular RNA-seq.

Supplementary Text S6: Perspective on HyperADARcd system

The original development in animals has comprehensively showed the advantages and limitations of ADARcd-based editing systems[14,26]. So far, this method has been successfully implemented in *in vitro* cultured *Drosophila* and mammalian cells as well as specific neuron cells from transgenic fly. Here we extended its use in plants including transient expression in *N. benthamiana* and transgenic expression in both Arabidopsis and rice. In comparison to aforementioned CLIP-derived methods, HyperADARcd system is apparently of simple-procedure, crosslinking-free, antibody-independent and cost-effective, which facilitates the RBP studies using a small amount of starting materials. We will underline the following perspective of this system.

There are a number of RBPs that are intrinsically inclined to forming phase separation condensates or even unreversible solid-like aggregates under the circumstances of genetic mutation, stress stimulation or even mild *in vitro* extraction variation. The former two conditions are biologically functional relevant. However, the current CLIPs count on *in vitro* IP with high-quality antibodies as a prerequisite. The efficiency and specificity largely requires tedious optimization, not merely the extraction buffer in our experience. Our study demonstrates that HyperADARcd system can authentically identify RNA species that are proximal to the RBP of interest in the phase separation condensates. Further, this system can also capture the dynamic RNA composition in the condensates as we detected altered target RNAs upon ETI induction (Fig. 1). We believe that this method will constitute a powerful strategy to study dynamic RNA associated with RBPs under both solvent and gel-like condensate conditions.

A further advantage of HyperADARcd system is that it does not require crosslinking and is therefore applicable to study target RNAs for both RBPs and RBP-associated proteins (RAPs). These RAPs may not directly contact with RNAs and its RAP-HyperADARcd will be directed to RNAs through their associated RBPs. RAPs are likely bearing a regulatory role, such as RACKs[51,52]. The same RAP may associate with different RBPs and vise versa. Understanding the intersecting and different interacting RNA pools of them will highlight a degree of molecular multitasking of their interplay in distinct RNP complexes.

In addition to the reported poly(A) RNA associated RBPs, future application of acidic guanidiniumthiocyanate-phenol-chloroformphase partition-based methods which were recently developed in animal systems will definitely add more non-poly(A) RNA associated RBP candidates to plant RBP proteome[53-55]. In fact, HyperADARcd system is still at an infant stage and quite a lot of improvements are required to sharpen it. Similar to the crosslinking bias in formaldehyde or UV applications, HyperADARcd displays editing bias towards a preferential editing motif (Fig. 1i). Alongside with this primary sequence preference, it also tends to edit preferential sites with a small RNA loop that can more capably dock HyperADARcd[26]. It means that after binding to RNA through RBPs, HyperADARcd still requires plenty of

time to search and edit the target site, the time window of which has been estimated to be around 24 seconds[56]. Therefore, elimination of preferences of the primary motif and secondary structure will definitely increase the catalytic rate of HyperADARcd. Crosslinking-based methods capture the snapshot of RBP-RNA interaction. In this sense, both long/strong and transient/weak interactions have been detected, the latter of which is biologically meaningful or not requires further proof. Nevertheless, optimization of HyperADARcd to one without preferences but with high editing rate close to crosslinking will be the focus at the next stage of the development. This task could draw on the experience of CRISPR development that takes the advantage of rapid directed protein evolution strategy[57].

Supplementary Text S7: Zero-background ligation-independent cloning of RBP fusion with HyperADARcd

- Primer design

  ➢ RBP-HyperADARcd conformation (constitutive vectors: pZG51 (basta), pZG56 (hygromycin); inducible vectors: pZG160 (basta), pZG163 (hygromycin)).

  Target-F: 5'- CGA CGA CAA GAC CGT ACC +++ (CDS w/ ATG)

  Target-R: 5'- GA GGA GAA GAG CCG TGC +++ (CDS w/o stop codon)

  ➢ HyperADARcd-RBP conformation (constitutive vectors: pZG165 (basta), pZG61 (hygromycin); inducible vectors: pZG159 (basta), pZG162 (hygromycin)).

  Target-F: 5'- C GAC GAC AAG ACC GTC +++ (CDS either w/ or w/o ATG)

  Target-R: 5'- GAG GAG AAG AGC CGT +++ (CDS w/ stop codon)

  ➢ HyperADARcd-RBP-HyperADARcd conformation (constitutive vectors: pZG66 (basta), pZG71 (hygromycin); inducible vectors: pZG161 (basta), pZG164 (hygromycin)).

  Target-F: 5'- CG ACG ACA AGA CCG TCC +++ (CDS either w/ or w/o ATG)

  Target-R: 5'- GA GGA GAA GAG CCG TGC +++ (CDS w/o stop codon)

- PCR amplification of RBP

- LIC-PCR fragment

Gel-purified PCR product	50 ng/kb (e.g. ~1 kb, add 50 ng)
10*Cutsmart buffer	1 µl
dATP (Promega, U1205)	0.5 µl
T4 DNA polymerase (NEB, M0203)	0.5 µl
H_2O	to 10 µl

12 ℃/30', 75 ℃/20'

- LIC-Vector

Plasmid	2 μg
10*Cutsmart buffer	10 μl
ApaI (NEB, R0114)	3 μl
H_2O	to 100 μl

25 ℃/60’, 65℃/20’

Then add:

dTTP (Promega, U1235)	5.6 μl
T4 DNA polymerase (NEB, M0203)	3 μl

12 ℃/30’, 75 ℃/20’

- LIC ligation (LIC-Vector : LIC-PCR fragment~1 : 3)

LIC-Vector	2.5 μl
LIC-PCR fragment	2.5 μl

75 ℃/5’, 22 ℃/10’

- Transformation and colony PCR

1. Use 1-2 μl ligation product to transfer ccdB sensitive *E.coli* strain, such as TOP10 and DH5α;
2. Then select on appropriate antibiotic (e.g., 50 μg/L kanamycin and 50 μg/L spectinomycin) LB plate. Perform colony PCR with Target-F/Target-R;
3. Sanger sequencing with appropriate primers.

Supplementary Text S8: References

1 Xu, G. *et al.* Global translational reprogramming is a fundamental layer of immune regulation in plants. *Nature* **545**, 487-490 (2017).

2 Hafner, M. *et al.* Transcriptome-wide identification of RNA-binding protein and microRNA target sites by PAR-CLIP. *Cell.* **141**, 129-141 (2010).

3 Licatalosi, D. D. *et al.* HITS-CLIP yields genome-wide insights into brain alternative RNA processing. *Nature.* **456**, 464-U422 (2008).

4 König, J. *et al.* iCLIP reveals the function of hnRNP particles in splicing at individual nucleotide resolution. *Nat Struct Mol Biol.* **17**, 909-915 (2010).

5 Weyn-Vanhentenryck, S. M. *et al.* HITS-CLIP and integrative modeling define the Rbfox splicing-regulatory network linked to brain development and autism. *Cell Rep.* **6**, 1139-1152 (2014).

6 Flynn, R. A. *et al.* Dissecting noncoding and pathogen RNA-protein interactomes. *RNA.* **21**, 135-143 (2015).

7 Zarnegar, B. J. *et al.* irCLIP platform for efficient characterization of protein-RNA interactions. *Nat Methods.* **13**, 489-+ (2016).

8 Van Nostrand, E. L. *et al.* Robust transcriptome-wide discovery of RNA-binding protein binding sites with enhanced CLIP (eCLIP). *Nat Methods.* **13**, 508-514 (2016).

9 Kargapolova, Y., Levin, M., Lackner, K. & Danckwardt, S. sCLIP-an integrated platform to study RNA-protein interactomes in biomedical research: identification of CSTF2tau in alternative processing of small nuclear RNAs. *Nucleic Acids Res.* **45**, 6074-6086 (2017).

10 Gu, J. *et al.* GoldCLIP: Gel-omitted Ligation-dependent CLIP. *Genomics Proteomics Bioinformatics.* **16**, 136-143 (2018).

11 Koster, T. & Meyer, K. Plant ribonomics: proteins in rearch of RNA partners. *Trends Plant Sci.* (2018).

12 Lin, C. Y. & Miles, W. O. Beyond CLIP: advances and opportunities to measure RBP-RNA and RNA-RNA interactions. *Nucleic Acids Res.* **47**, 5490-5501 (2019).

13 Ramanathan, M., Porter, D. F. & Khavari, P. A. Methods to study RNA–protein interactions. *Nat Methods.* **16**, 225-234 (2019).

14 McMahon, A. C. *et al.* TRIBE: Hijacking an RNA-editing enzyme to identify cell-specific targets of RNA-binding proteins. *Cell.* **165**, 742-753 (2016).

15 Mili, S. & Steitz, J. A. Evidence for reassociation of RNA-binding proteins after cell lysis: implications for the interpretation of immunoprecipitation analyses. *RNA.* **10**, 1692-1694 (2004).

16 Riley, K. J., Yario, T. A. & Steitz, J. A. Association of argonaute proteins and microRNAs can occur after cell lysis. *RNA.* **18**, 1581-1585 (2012).

17 Biswas, J., Rahman, R., Gupta, V., Rosbash, M. & Singer, R. H. MS2-TRIBE evaluates both protein-RNA interactions and nuclear organization of transcription by RNA editing. *iScience* **23** (2020).

18 Riley, Kasandra J. & Steitz, Joan A. The “observer effect” in genome-wide surveys of protein-RNA interactions. *Mol Cell.* **49**, 601-604 (2013).

19 Van Nostrand, E. L. *et al.* A large-scale binding and functional map of human RNA-binding proteins. *Nature.* **583**, 711-719 (2020).

20 Darnell, R. B. HITS-CLIP: panoramic views of protein–RNA regulation in living cells. *Wiley Interdiscip Rev RNA.* **1**, 266-286 (2010).

21 Lambert, N. *et al.* RNA Bind-n-Seq: quantitative assessment of the sequence and structural binding specificity of RNA binding proteins. *Mol Cell.* **54**, 887-900 (2014).

22 Kim, B. & Kim, V. N. fCLIP-seq for transcriptomic footprinting of dsRNA-binding proteins: Lessons from DROSHA. *Methods.* **152**, 3-11 (2019).

23 Lapointe, C. P., Wilinski, D., Saunders, H. A. J. & Wickens, M. Protein-RNA networks revealed through covalent RNA marks. *Nat Methods.* (2015).

24 Fazal, F. M. *et al.* Atlas of subcellular RNA localization revealed by APEX-seq. *Cell.* **178**, 473 (2019).

25 Medina-Munoz, H. C., Lapointe, C. P., Porter, D. F. & Wickens, M. Records of RNA locations in living yeast revealed through covalent marks. *Proc Natl Acad Sci USA.* **117**, 23539 (2020).

26 Rahman, R., Xu, W., Jin, H. & Rosbash, M. Identification of RNA-binding protein targets with HyperTRIBE. *Nat Protoc.* **13**, 1829-1849 (2018).

27 Gal-Mark, N., Schwartz, S., Ram, O., Eyras, E. & Ast, G. The pivotal roles of TIA proteins in 5' splice-site selection of alu exons and across evolution. *PLoS Genet.* **5**, e1000717 (2009).

28 Dember, L. M., Kim, N. D., Liu, K. Q. & Anderson, P. Individual RNA recognition motifs of TIA-1 and TIAR have different RNA binding specificities. *J Biol Chem.* **271**, 2783-2788 (1996).

29 Lambermon, M. H. L. *et al.* UBP1, a novel hnRNP-like protein that functions at multiple steps of higher plant nuclear pre-mRNA maturation. *EMBO J.* **19**, 1638-1649 (2000).

30 Lambermon, M. H. L. *et al.* UBA1 and UBA2, two proteins that interact with UBP1, a multifunctional effector of pre-mRNA maturation in plants. *Mol Cell Biol.* **22**, 4346-4357 (2002).

31 Del Gato-Konczak, F. *et al.* The RNA-binding protein TIA-1 is a novel mammalian splicing regulator acting through intron sequences adjacent to a 5 ' splice site. *Mol Cell Biol.* **20**, 6287-6299 (2000).

32 Sorenson, R. & Bailey-Serres, J. Selective mRNA sequestration by OLIGOURIDYLATE-BINDING PROTEIN 1 contributes to translational control during hypoxia in Arabidopsis. *Proc Natl Acad Sci USA.* **111**, 2373-2378 (2014).

33 Meyer, C. *et al.* The TIA1 RNA-binding protein family regulates EIF2AK2-mediated stress response and cell cycle progression. *Mol Cell.* **69**, 622-635 e626 (2018).

34 Rayman, J. B., Karl, K. A. & Kandel, E. R. TIA-1 self-multimerization, phase separation, and recruitment into stress granules are dynamically regulated by Zn2+. *Cell Rep.* **22**, 59-71 (2018).

35 Weber, C., Nover, L. & Fauth, M. Plant stress granules and mRNA processing bodies are distinct from heat stress granules. *Plant J.* **56**, 517-530 (2008).

36 Nguyen, C. C. *et al.* Oligouridylate binding protein 1b plays an iIntegral role in plant heat stress tolerance. *Front Plant Sci.* **7** (2016).

37 Nguyen, C. C. *et al.* Overexpression of oligouridylate binding protein 1b results in ABA hypersensitivity. *Plant Signal Behav.* **12**, 0 (2017).

38 Boutrot, F. & Zipfel, C. Function, discovery, and exploitation of plant pattern recognition receptors for broad-spectrum disease resistance. *Annu Rev Phytopathol.* **55**, 257-286 (2017).

39 Liang, X. & Zhou, J. M. Receptor-like cytoplasmic kinases: central players in plant receptor kinase-mediated signaling. *Annu Rev Plant Biol.* **69**, 267-299 (2018).

40 Li, B., Meng, X., Shan, L. & He, P. Transcriptional regulation of pattern-triggered immunity in plants. *Cell Host Microbe.* **19**, 641-650 (2016).

41 Kanyuka, K. & Rudd, J. J. Cell surface immune receptors: the guardians of the plant's extracellular spaces. *Curr Opin Plant Biol.* **50**, 1-8 (2019).

42 Jones, J. D., Vance, R. E. & Dangl, J. L. Intracellular innate immune surveillance devices in plants and animals. *Science.* **354** (2016).

43 Lapin, D., Bhandari, D. D. & Parker, J. E. Origins and immunity networking functions of EDS1 family proteins. *Annu Rev Phytopathol.* **58**, 253-276 (2020).

44 Ngou, B. P. M., Ahn, H. K., Ding, P. & Jones, J. D. G. Mutual potentiation of plant immunity by cell-surface and intracellular receptors. *Nature.* (2021).

45 Yuan, M. *et al.* Pattern-recognition receptors are required for NLR-mediated plant immunity. *Nature.* (2021).

46 Thomma, B. P., Nurnberger, T. & Joosten, M. H. Of PAMPs and effectors: the blurred PTI-ETI dichotomy. *Plant Cell.* **23**, 4-15 (2011).

47 Ntoukakis, V., Saur, I. M., Conlan, B. & Rathjen, J. P. The changing of the guard: the Pto/Prf receptor complex of tomato and pathogen recognition. *Curr Opin Plant Biol.* **20**, 69-74 (2014).

48 Tang, X. *et al.* Initiation of plant disease resistance by physical interaction of *AvrPto* and *Pto* kinase. *Science.* **274**, 2060-2063 (1996).

49 Pedley, K. F. & Martin, G. B. Molecular basis of Pto-mediated resistance to bacterial speck disease in tomato. *Annu Rev Phytopathol.* **41**, 215-243 (2003).

50 Ramos, R. N., Martin, G. B., Pombo, M. A. & Rosli, H. G. WRKY22 and WRKY25 transcription factors are positive regulators of defense responses in *Nicotiana benthamiana*. *Plant Mol Biol.* **105**, 65-82 (2021).

51 Li, J. J. & Xie, D. RACK1, a versatile hub in cancer. *Oncogene.* **34**, 1890-1898 (2015).

52 Su, J., Xu, J. & Zhang, S. RACK1, scaffolding heterotrimeric G protein and MAPK cascade. *Trends Plant Sci.* (2015).

53 Queiroz, R. M. L. *et al.* Comprehensive identification of RNA–protein interactions in any organism using orthogonal organic phase separation (OOPS). *Nat Biotechnol.* **37**, 169-178 (2019).

54 Trendel, J. *et al.* The human RNA-binding proteome and its dynamics during translational arrest. *Cell* **176**, 391-403.e319 (2019).

55 Urdaneta, E. C. *et al.* Purification of cross-linked RNA-protein complexes by phenol-toluol extraction. *Nat Commun.* **10**, 990 (2019).

56 Kuttan, A. & Bass, B. L. Mechanistic insights into editing-site specificity of ADARs. *Proc Natl Acad Sci USA.* **109**, E3295 (2012).

57 Packer, M. S. & Liu, D. R. Methods for the directed evolution of proteins. *Nat Rev Genet.* **16**, 379-394 (2015).

Supplementary Figures

Fig. S1

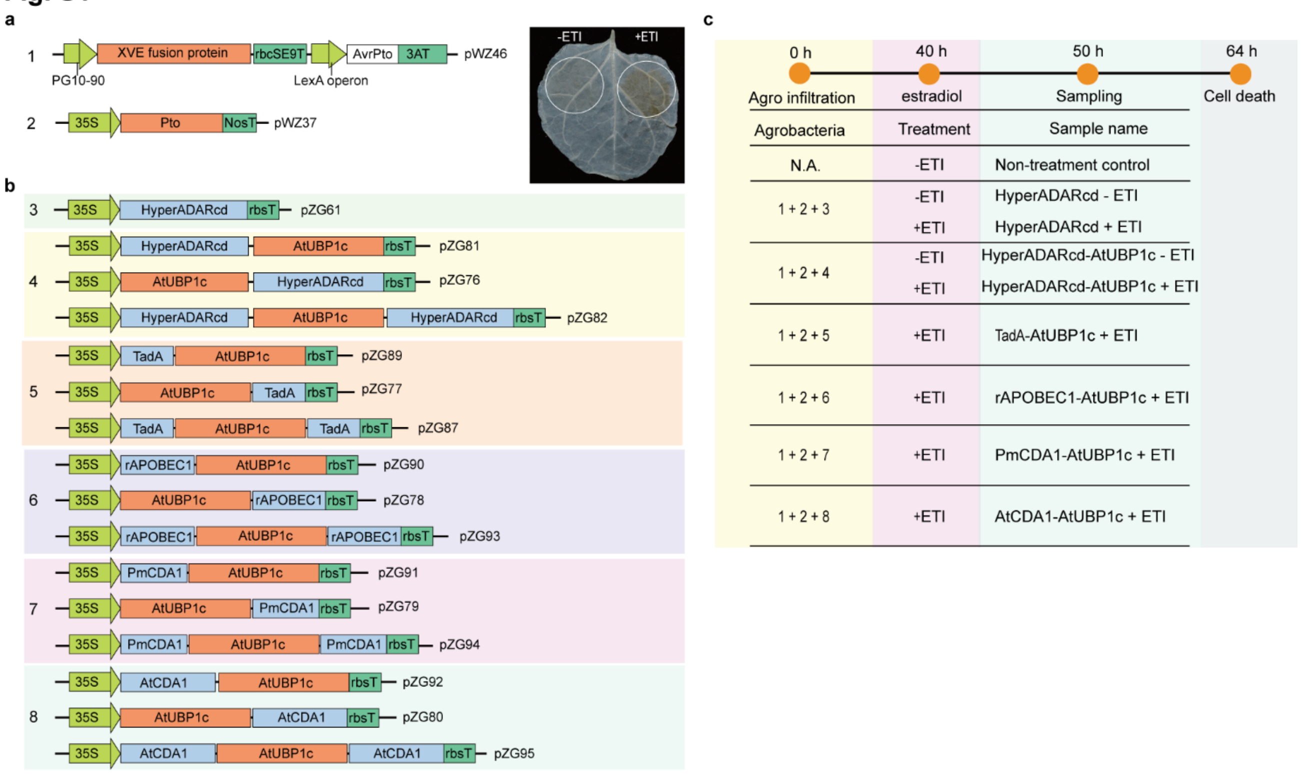

Supplementary Fig. S1. ETI induction and schematic of sample preparation. a, ETI induction in *N. benthamiana* by recognition between β-estradiol-controlled bacterial effector AvrPto (1) and the corresponding constitutively-expressed host target Pto (2). Application of β-estradiol promotes the translocation of XVE fusion protein into nucleus where it binds to LexA operon and drives the transcription of AvrPto, leading to ETI activation with spontaneous cell death (+ETI) in comparison to application of water (-ETI). Leaves were decolorized using ethanol. **b**, Schematic of vectors used in transient assays. HyperADARcd alone (3) was used as a control. AtUBP1c was fused with each RNA editing enzyme in three different conformations. **c**, A time window for sampling. Agrobacteria containing the indicated constructs were transiently expressed in *N. benthamiana* for 40 h and treated with (+ETI) or without (-ETI) estradiol for another 10 h when no macroscopic cell death occurred. Sample names are used as legends in all figure presentation.

Fig. S2

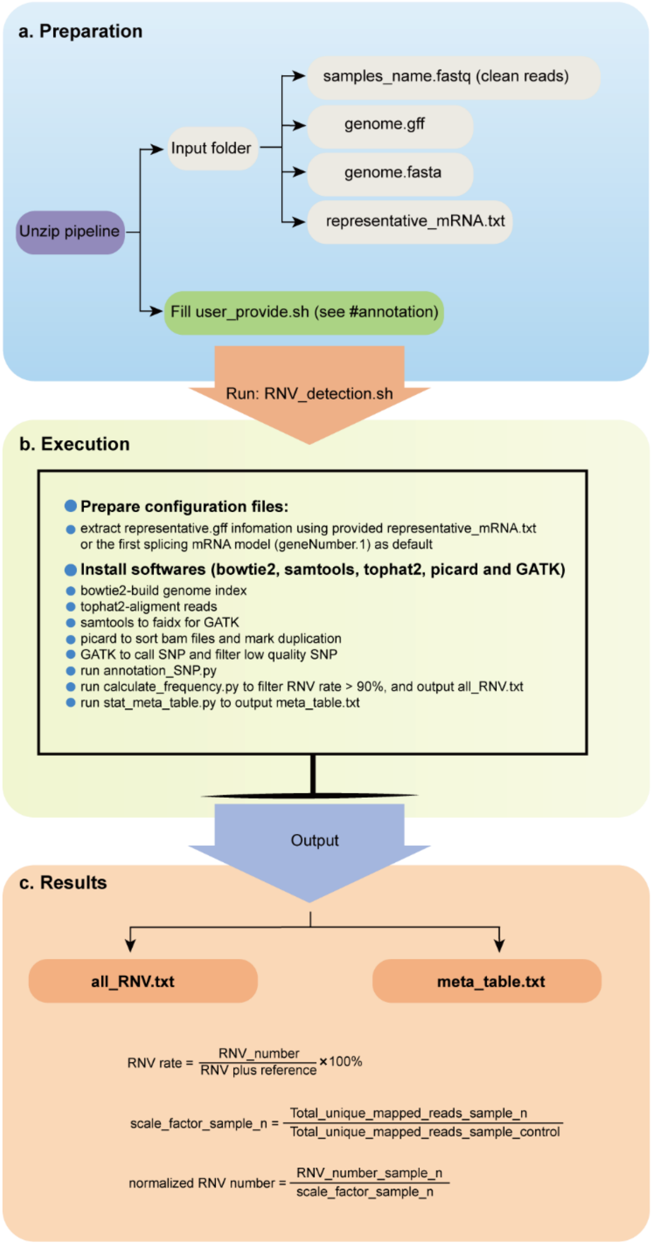

Supplementary Fig. S2. RNV detection pipeline. a-c, The pipeline contains three parts of Preparation (**a**, blue), Execution (**b**, yellow) and Results (**c**, orange). Users unzip the downloaded pipeline package from http://uorflight.whu.edu.cn/public/ to their working directory and put files of samples_name.fastq (clean reads for all samples), genome.fasta, genome.gff and representative_mRNA.txt (optional) into the 'Input' folder. File information is required to fill into user_provided.sh following the #annotation (**a**). Running RNV_detection.sh will automatically install the required packages (requiring user's permission on the server computer) and return two tables to the 'Results' folder (**b**). The all_RNV.txt table contains RNV information of chromosome, transcript name, etc. The other meta_table.txt table contains summary information for each sample, including total unique mapped reads for calculation of scale_factor_sample_n and 12-type RNV numbers (**c**). See Supplementary Table S2 for examples of these two tables. Scale factors are used to normalize

RNV numbers for comparison among different RNA-seq sample libraries. Read README in the package for troubleshooting.

Fig. S3

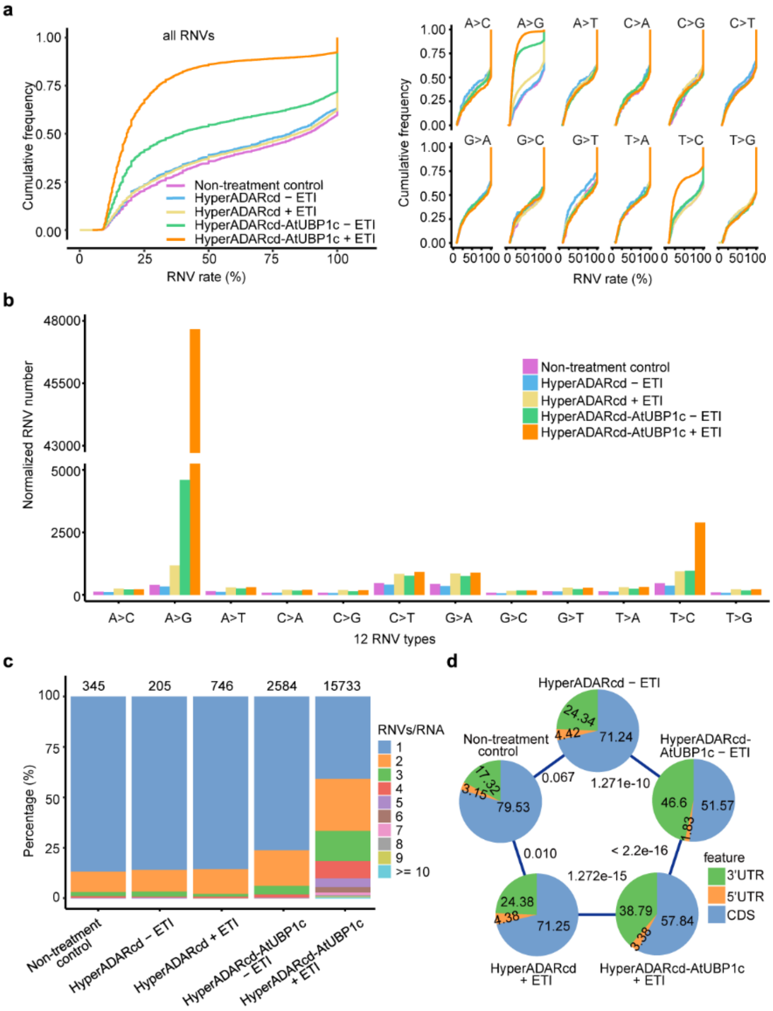

Supplementary Fig. S3. Characterization of RNV detection by transient expression in *N. benthamiana*. **a**, Cumulative frequency curve showing the RNV rate distribution patterns of all RNVs and each of the 12-type RNVs. **b**, The total number of each of the filtered 12-type RNVs (removing RNVs with rate > 90%). **c**, Percentage of different levels of RNV number per RNA molecule (RNVs/RNA). Total RNV numbers in each sample are indicated above. **d**, Pie chart of A>G RNV distribution on different mRNA features. Data are shown as percentage of A>G RNV in each feature. Pearson's chi-squared test. See Fig. 1 for more information.

Fig. S4

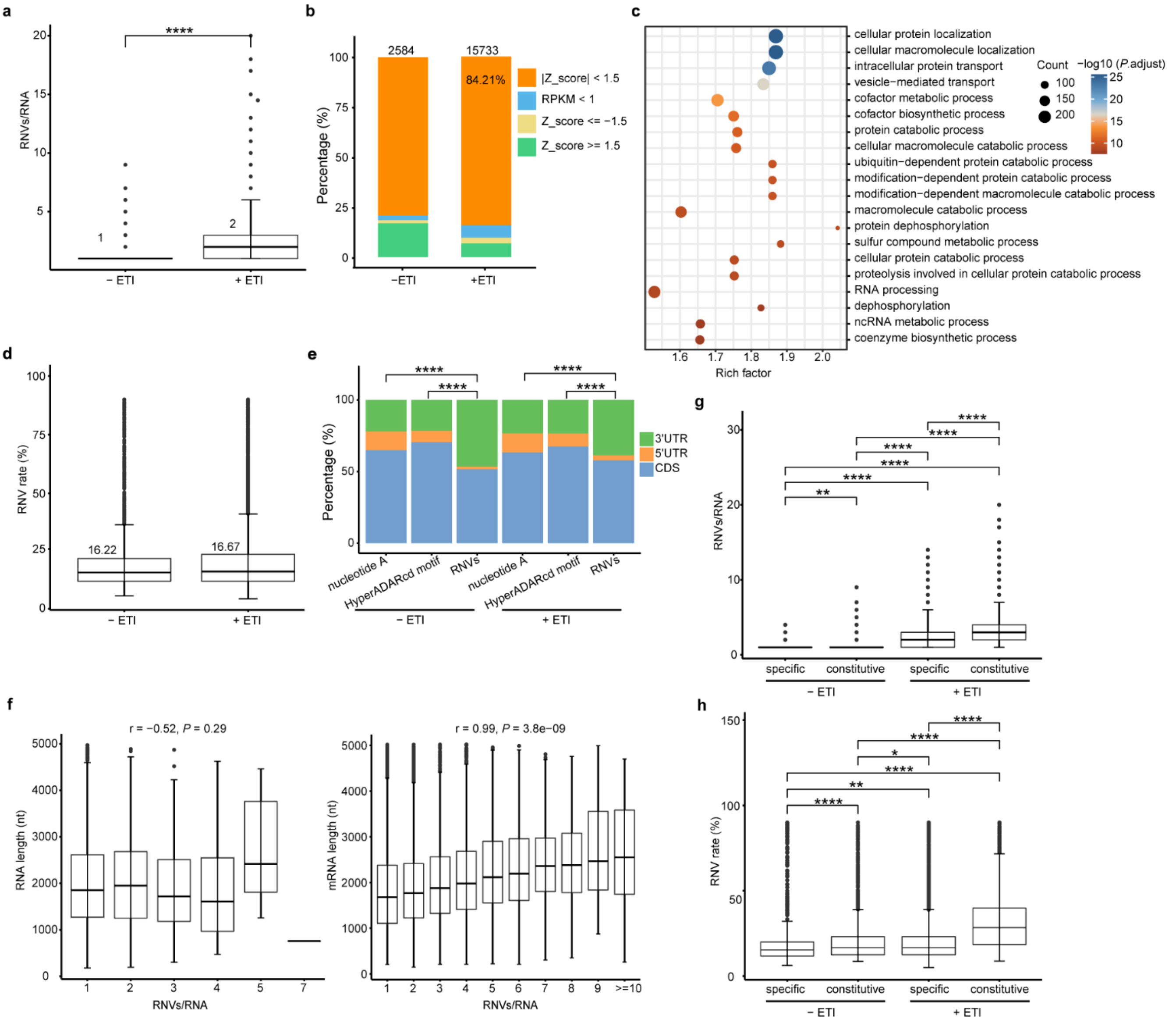

Supplementary Fig. S4. ETI effects on the behaviors of AtUBP1c-HyperADARcd in *N. benthamiana*. a, Boxplot showing distribution of RNVs/RNA for -ETI and +ETI targets. Values above the box mean median. Wilcoxon rank-sum test. **b**, Percentage of different levels of RNA-seq fold changes (RSfc). Target genes with RNA level ≥ 1 RPKM were further divided into three groups using Z_score method. Total gene numbers of -ETI and +ETI targets are indicated above. **c**, Gene Ontology (GO) analysis of +ETI target genes. **d**, Boxplot to show the distribution of RNV rate for -ETI and +ETI targets. Values above the box mean median. **e**, Relative abundance of RNV distribution, compositions of nucleotide A and HyperADARcd preferential motif on different RNA features. Pearson's chi-squared test. **f**, Relationship of RNVs/RNA with RNA length for -ETI (left) and +ETI (right) targets. Pearson correlation coefficient r and significance *P* are shown for RNVs/RNA with different median of RNA length. **g**, **h**, Boxplot showing distribution of RNVs/RNA (**g**) and RNV rate (**h**) for specific and constitutive groups of -ETI and +ETI targets. Kruskal-Wallis test before post-hoc analysis

with Dunn’s test was used. For all boxplots, center line, median; box limits, upper and lower quartiles; whiskers, 1.5 × interquartile range; points, outliers.

Fig. S5

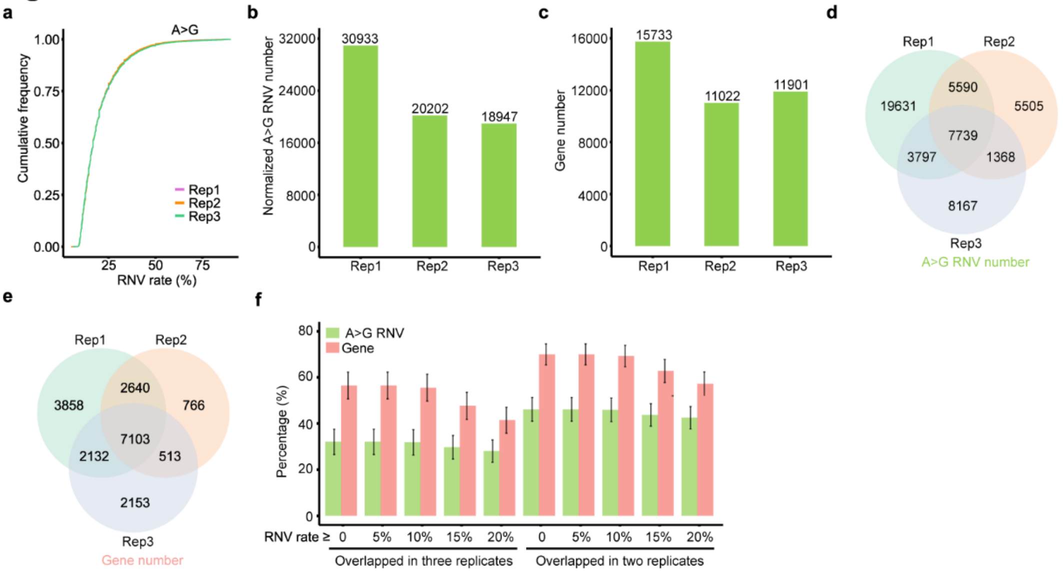

Supplementary Fig. S5. Reproducibility of HyperADARcd system in three independent experimental replicates. Two independent experiments (Rep2/3) of HyperADARcd-AtUBP1c upon ETI activation in *N. benthamiana* are compared with Rep1 used in Fig. 1. **a**, Cumulative frequency curve showing the RNV rate distribution pattern of A>G RNV. **b**, **c**, The total normalized number of filtered A>G RNVs (**b**) and target genes (**c**) in each replicate. **d**, **e**, Venn diagram showing the overlapping of filtered A>G RNVs (**d**) and target genes (**e**) in each replicate. **f**, Summary of the overlapping percentage of A>G RNVs (green) and target genes (pink) in three or any two replicates with changed thresholds of RNV rate. Data are represented as means ± SD of the percentage of overlapped fractions in each replicates.

Fig. S6

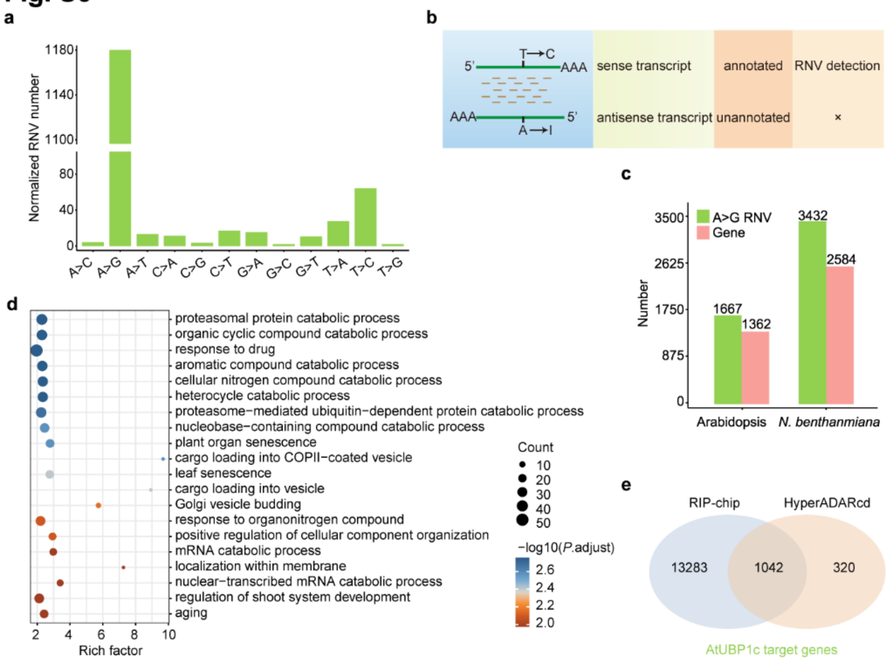

Supplementary Fig. S6. Comparison of AtUBP1c target RNAs with the public data. Three-week-old transgenic Arabidopsis T1 lines were used to induce the expression of HyperADARcd-AtUBP1c using β-estradiol for 10 h. Samples were combined from 10 individual lines for RNV detection. **a**, The total number of each of the filtered 12-type RNVs. **b**, Schematic overview showing the detection of unexpected T>C RNV. Non-stranded RNA-seq library could not distinguish the reads from either sense or antisense transcripts. A>G editing on antisense transcripts which usually exist in a small amount of the transcriptome leads to T>C in the annotated sense strand. **c**, A>G RNV (green) and gene (pink) numbers of HyperADARcd-AtUBP1c detected in transgenic Arabidopsis or transient expression in *N. benthamiana* (Sample HyperADARcd-AtUBP1c-ETI in Fig. 1). **d**, Gene Ontology (GO) analysis of HyperADARcd-AtUBP1c target RNAs in transgenic Arabidopsis. **e**, Venn diagram showing the overlapping of AtUBP1c target RNAs detected by HyperADARcd system and by RNA>Immunoprecipitation following with microarray detection (RIP-chip) in the literature (Sorenson and Bailey-Serres, 2014, PNAS).

Fig. S7

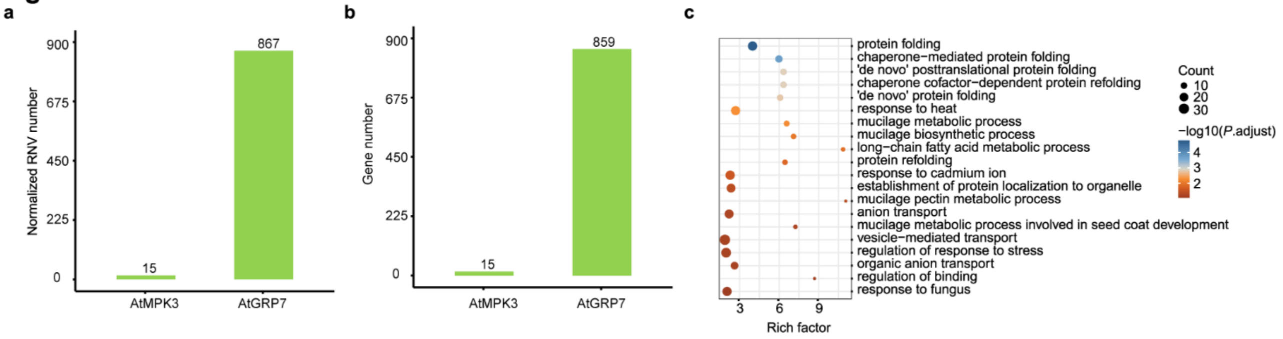

Supplementary Fig. S7. Validation of HyperADARcd system using non-RBP and RBP. Three-week-old transgenic Arabidopsis T1 lines were used to induce the expression of non-RBP fusion (HyperADARcd-AtMPK3) and RBP-fusion (HyperADARcd-AtGRP7), respectively, using β-estradiol for 10 h. Each sample was combined from 10 individual lines for RNV detection. **a**, **b**, The total number of filtered A>G RNVs (**a**) and target genes (**b**) in each sample. **c**, Gene Ontology (GO) analysis of HyperADARcd-AtGRP7 target RNAs in transgenic Arabidopsis.

Fig. S8

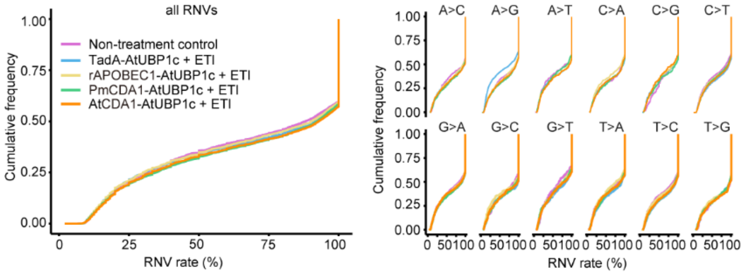

Supplementary Fig. S8 Characterization of other RNA editing enzymes in RNV detection during ETI activation. AtUBP1c fused with TadA, rAPOBEC1, PmCDA1 and AtCDA1 were transiently expressed in *N. benthamiana* and treated by ETI induction. Cumulative frequency curve showing the RNV rate distribution patterns of all RNVs and each of the 12-type RNVs.

Fig. S9

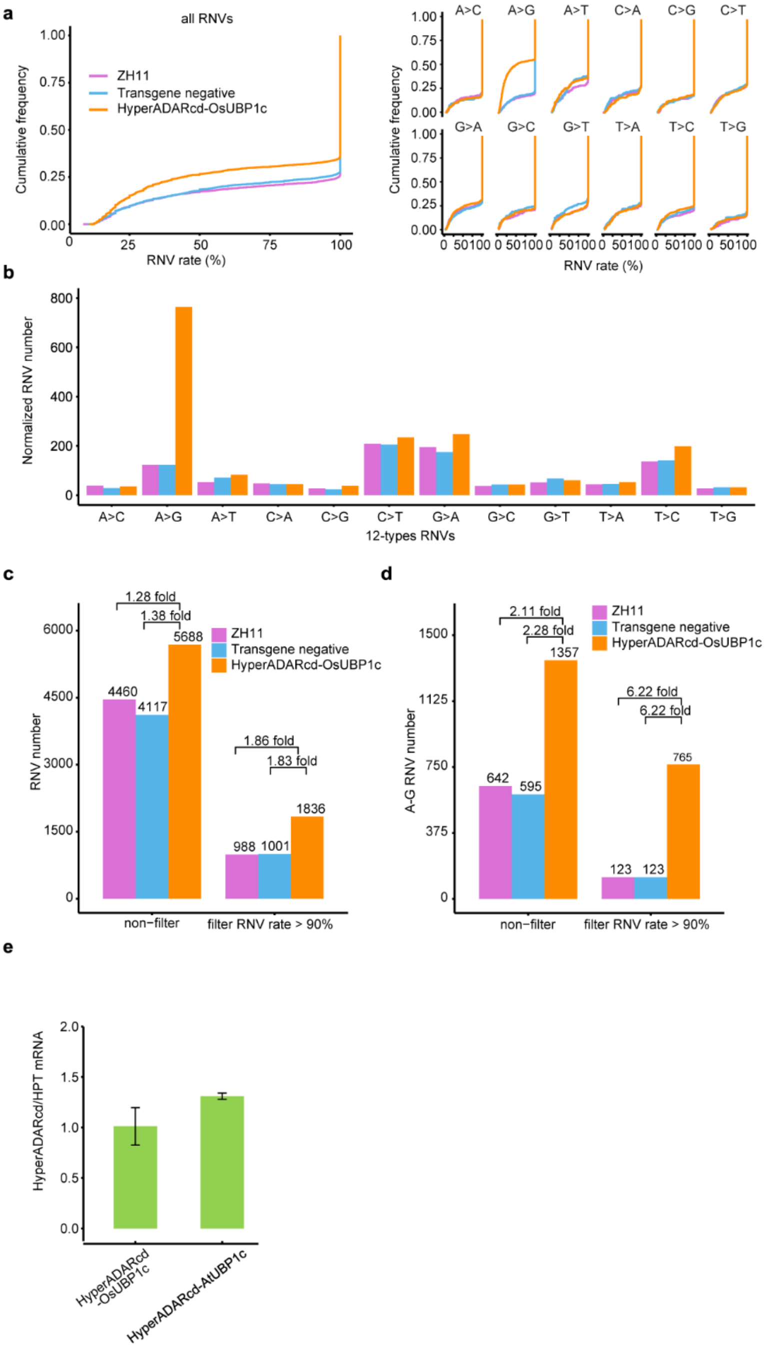

Supplementary Fig. S9. Characterization of RNV detection in transgenic rice. OsUBP1c-HyperADARcd transgenic T0 plants are compared to non-transgenic parent ZH11 and transgene negative progeny controls. **a**, Cumulative frequency curve showing the RNV rate distribution patterns of all RNVs and each of the 12-type RNVs. **b**, The total number of each of the filtered 12-type RNVs (removing RNVs with rate > 90%). **c**, **d**, Effects of filtering RNVs with rate > 90% on identification of total RNVs (**c**) and A>G RNVs (**d**). **e**, Quantitative PCR showing the expression of HyperADARcd in *N. benthamiana* and transgenic rice. Data are represented as means ± SD (n = 3) of HyperADARcd mRNA with hygromycin resistance gene expressed from the same vector backbone as an internal control.

Fig. S10

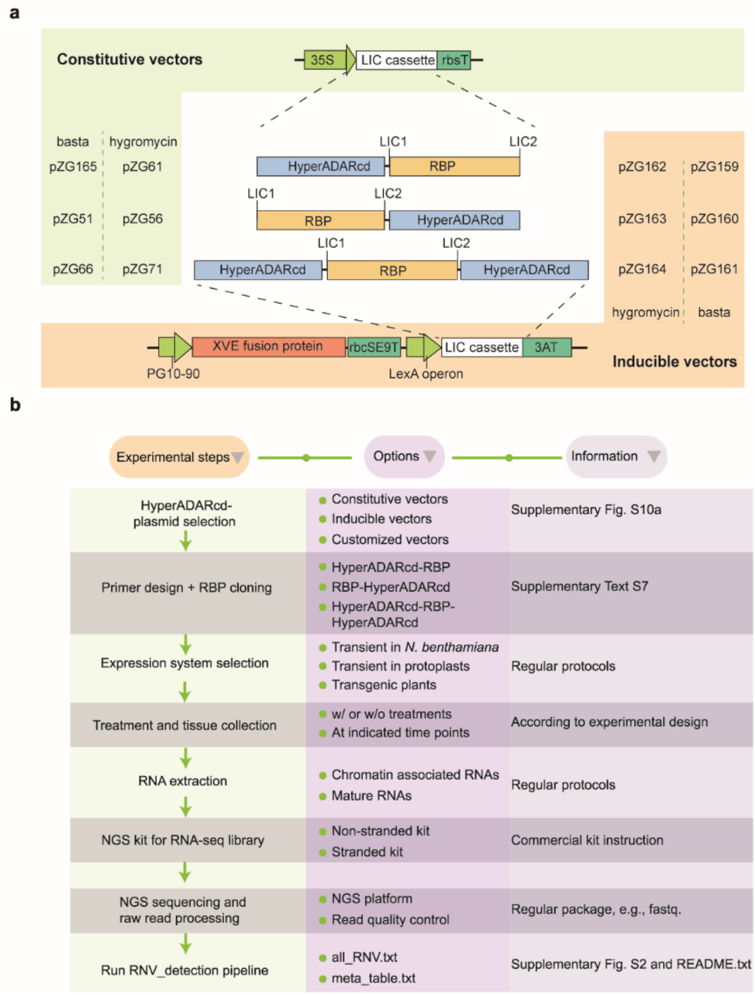

Supplementary Fig. S10. Schematic overview of constitutive and inducible expression vectors, and the workflow of HyperADARcd system in plants. a, Constitutive vectors drive the expression of LIC cassette by CaMV 35S promoter. Inducible vectors drive the expression of LIC cassette by β-estradiol controlled LexA operon. NosT, rbcSE9T and 3AT terminators are from *Nos*, pea *rbsS E9* and pea *3A* genes. LIC cassette is designed to express HyperADARcd and RBP fusion in three conformations using zero-background ligation-independent cloning (LIC) strategy (see Supplementary Text Sfor the detailed protocol). All conformations in constitutive and inducible vectors contain plant selection markers of either basta or hygromycin. See Supplementary Table S6 for more information, e.g., GenBank accession number. **b**, The workflow schematic of HyperADARcd system in plants. It contains three parts, experimental steps and the matching options in conjunction with the needed information. HyperADARcd-RBP fusion proteins can be expressed from plasmids generated in this study (**a**) or from customized plasmids such as replacing CaMV 35S promoter with native promoters. When continuing with LIC-based plasmids in this study, users are recommended to following the step-by-step cloning procedure described in Supplementary Text S7. HyperADARcd system has been tested in transient expression in *N. benthamiana* and transgenic expression in Arabidopsis and rice, and likely it will work in other expression systems such as protoplasts. According to the experimental design,

users can perform different treatments and collect tissues at the indicated time points. Since some RBPs bind to intron or other elements on newly transcribed RNAs, users are suggested to follow the regular protocols for isolation of either chromatin-associated native RNAs or mature RNAs. RNA-seq library construction can be performed using either relative inexpensive non-stranded commercial kits or stranded kits, the latter of which can reserve the direction information of RNA on the sequencing reads. Quality control and processing of the raw sequencing reads are done using publicly popular pipelines, such as Fastq. The clean reads are transferred into our RNV_detection pipeline by following the instruction depicted in Supplementary Fig. S2. Users can generate their desired figures using the resulting files from our pipeline.
